# Relationship between Hedonic Hunger and Health Interest on Habit and Sodium Intake Patterns in Food Consumption

**DOI:** 10.1155/2019/9517140

**Published:** 2019-07-22

**Authors:** Imam Santoso, Susinggih Wijana, Afita Ismawati, Wenny Bekti Sunarharum

**Affiliations:** ^1^Agroindustrial Management Laboratory, Faculty of Agricultural Technology Universitas Brawijaya, Malang, Indonesia; ^2^Creative Agroindustry Research group, Universitas Brawijaya, Malang, Indonesia; ^3^Agro-chemistry Technology Laboratory, Faculty of Agricultural Technology, Universitas Brawijaya, Malang, Indonesia; ^4^Sensory and Applied Food Science Research Group, Faculty of Agricultural Technology, Universitas Brawijaya, Malang, Indonesia

## Abstract

Many people are motivated to eat healthily but find it difficult to override established and less healthy habits. Habits, by their nature, are unconscious and cued by the environment, thus making them powerful determinants of behavior. This study examined how hedonic hunger and health interest are related to habit and whether sodium consumption is mediated by hedonic hunger, health interest, and habit. A total of 117 students of Universitas Brawijaya took part in the study. Data analysis were done using Partial Least Square (PLS) and a semi-quantitative food frequency questionnaire (SQ-FFQ). PLS was used to analyze the influence of the relationship between independent and dependent variables. SQ-FFQ was used to determine sodium intake in grams/day. The average sodium intake in this study was 2.47 grams/day. This analysis shows that hedonic hunger and health interest had a significant impact on health habits but not on sodium intake.

## 1. Introduction

Consumers often mention that health considerations are one of the motivations for choosing particular foods [1]. Adequate nutrition intake is an important aspect of leading a healthy life [2]. The importance of health is important to some people when choosing which food to consume [3]. Misguided diet or inappropriate food selection can be factors in developing various diseases such as high blood pressure (hypertension). Research shows that people who are very motivated to meet their health goals form healthy eating habits and do not have to struggle against unwanted desires [4].

Based on [5], every year, 9.4 million people risk death from heart disease and stroke because of high blood pressure. If these two diseases are combined, they are the number one cause of death in the world. Hypertension also increases the risk of kidney failure, blindness, and other diseases and often coincides with other health risk factors such as obesity, diabetes high cholesterol. Interestingly, WHO reported that high-income countries had lower rates of hypertension compared to low to middle-income countries. Based on Indonesian's Ministry of Health data in 2013, 25.8% of its population suffered from hypertension. In Indonesia, there has been a shift in eating patterns to the consumption of fast food and preserved foods. This type of consumption pattern has developed very quickly, especially in major cities. It is known that fast food and preserved food contain high salt, saturated fats also low in fat that can cause hypertension.

One of the high risk factors in developing hypertension is increased salt intake [6]. Salt is a source of sodium [7]. Sodium is not only found in table salt, but also found naturally in most foods, including milk, cream, eggs, meat, and clams. High quantities of sodium are also found in processed foods such as bread, crackers; processed meats, such as bacon, sausages and ham; snacks such as popcorn; and spices such as soy sauce, fish sauce and broth [8]. According to [9], people are often unaware of the amount of salt they consume. Uncontrolled sodium intake is caused by casual dietary consumption patterns such as habit, hedonic hunger and health interest. Decreasing sodium intake is proven to significantly reduce blood pressure in adults.

Habit can be defined as a psychological tendency to repeat past behavior [10]. Habits are certain behaviors that a person does repeatedly in a fixed situation to pursue his or her goals [11]. Although habit formation allows individuals to perform his or her daily routines in a very efficient way, habits also have negative consequences. Several studies have shown that people find it difficult to eliminate unwanted habits; one of them is the habit of eating everyday foods [12–14]. Habit plays an important role in maintaining the appropriate level of nutrition in individuals of all ages [15].

The habit of consuming salty food results in high level of sodium in the body. That habit can be minimized by dieting to control the daily food intake. Diet is considered as one of the most important determinants in controlling heart disease and risk factors as associated with hypertension and obesity. Evidence-based data shows that an effective diet regime is important in the relationship between health and illness [16]. The habit of choosing healthy foods also protects individuals against the desire to eat large amounts of food or unhealthy foods [4].

Hedonic hunger is defined as the driver for people to consume food when they are not really hungry, for example, consuming a dessert due to its pleasurable experience [17]. The hedonic driver from today's environment provides a wide selection of very cheap tasty food [18]. As a result, hedonic hunger can be a stimulus for food consumption contradicting to a person's desire for a healthy diet [19]. Hedonist behavior also causes the consumer to make too many dietary mistakes when choosing food because of their inability to control their food intake [20].

Unhealthy eating behavior is also determined by the health interest of the consumer. Diet has been shown to be associated with various diseases such as obesity, heart disease, cancer type 2 diabetes. The main dietary concerns include over consumption of saturated and trans fats and low consumption of vegetables fruits and grains. Today's food is very varied in taste and price. It is easy for consumers to choose a healthy diet especially as there is growing evidence that the easy availability of healthy foods has an important influence on food choice [21]. However, only health-conscious individuals are concerned about the importance of a healthy diet and change the diet accordingly to benefit from healthy products [22]. The choice of a healthy diet is associated with the health risks that people face and it is strongly affected by the health behaviors adopted [23].

Based on the explanations described above, this study aims to determine the relationship between hedonic hunger and health interest against habit. In addition, this study also aims to determine the relationship between hedonic hunger, health interest, and sodium intake patterns.

## 2. Method

### 2.1. Population and Sample

The base population of this study consisted of the students from the Universitas Brawijaya. The data used is from 2014, collected from 135 sample students out of the 55,000 students at the Universitas Brawijaya. The sampling method employed Slovin formula which has an error margin of 10%, thus at least 100 participants were needed; 135 students were selected to participate in this research. The sampling technique was done randomly so all members of the parent population had equal opportunity to be in the sample [24].

### 2.2. Measurement

The questionnaire was prepared specifically for this study and adjusted to measure sodium consumption, habit, hedonic hunger and health interests. The item for each question was a modification of previous research and was adapted to the purpose of this study, which was to determine the salt consumption of the participants. The hedonic hunger questionnaire was adapted from The Power of Food Scale (PFS). PFS is a self-made measurement in assessing the extent to which appetizing foods affect one's thoughts and feelings when they are not physically hungry [25]. The hedonic hunger questionnaire consisted of 10 questions on three levels of food proximity, the desire to eat something but not physically present for consumption, food present but not eaten and food tasted but not consumed [12, 25]. Health interest was determined by using 5 questions based on GHI (General Health Interest) adapted from Fenko's 2015 research which concerned the content of healthy food such as protein, nutrients and minerals. Habit was measured by using 7 questions that represented two habits of behavior, repetitive and automatic with regard to consuming salty food. The complete question set is given in Table 1.

The daily consumption pattern of sodium was measured by using SQ-FFQ (Semi Quantitative Food Frequency Questionnaire), which is a common method applied for estimation of dietary intake based on long term food intake in large epidemiological studies [26, 27]. Measurement of sodium intake was adapted from salt intake questionnaire of [28] and dietary sodium intake of [29] that had been tested and validated. A total of 34 items were used to measure daily sodium intake. To calculate the sodium intake, participants estimated how many times a particular had been food consumed in the last 3 months. Food consumption was measured into four categories: never, daily, weekly, and monthly. In the daily category, respondents were asked to state how many times a food was consumed in a day; similarly for weekly and daily consumption.

### 2.3. Data Analysis

The data analysis method employed in this research was descriptive and inferential. Descriptive analysis was used to explain the general description that occurred to the respondents. The software used for descriptive analysis was SPSS 18.0 and NutriSurvey 2007. SPSS was used to analyse the general descriptions given by the respondents. NutriSurvey 2007 was used to determine the nutritional content of a food and determine sodium intake in this study. Inferential analysis was used to perform the conceptual test stated in the research hypothesis. In accordance with the hypothesis that had been formulated, the method used in this study was Partial Least Square (PLS) which is an alternative approach that shifts the covariance-based Structural Equation Modeling (SEM) approach to variance-based one. SEM generally tests the causality while PLS is more predictive.

## 3. Results

### 3.1. Description of Respondents' Characteristics

There were 135 questionnaires distributed in this study. However, only 117 questionnaires were considered valid. The results of sodium consumption based on respondents' characteristics can be seen in Table 2. Table 2 shows the mean and standard deviation of sodium consumption (gram/day).

Based on Table 2, it can be seen that the average sodium consumed by men was 2.49 gram/day, while the average sodium consumed by women was 2.46 gram/day. The average sodium consumption consumed by respondents aged 16-20 year old was 2.761 gram/day, while the average sodium consumed by respondents aged 21-25 year old was 2.37. Based on the results of this study, the consumption of sodium of respondents at the age of 16-20 year old was greater than the respondents aged 21-25 year old. Sodium consumption for respondents whose monthly expenses were less than 1 million was 2.52 gram/day. As for the respondents who spent 1-5 million, the sodium consumption was 2.40 gram/day. The respondents in this study only consisted of two BMI categories, underweight and normal weight. The sodium intake for normal weight was 2.55 gram/day, while for the underweight category was 2.24 gram/day. This study indicated that sodium consumption of normal weight people was higher than underweight people. It was contrary to the findings of [30] who showed that there was a significant relationship between sodium increase and BMI increase. The results also revealed that the average sodium consumed per individual was 2.48 gram/day; it was not in accordance with the WHO recommendation which recommended a sodium intake of less than 2 gram/day or 5 gram of salt per day [31].

### 3.2. Results of Data Analysis Using Partial Least Square

#### 3.2.1. Result of Measurement Model Evaluation

Outer loadings, convergent validity, average variance extracted (AVE=discriminant validity) and composite reliability (CR) were used to test the reflective model measurement [32]. The result of reflective measurement model evaluation can be seen in Table 3.

Convergence validity test for reflective indicator used loading value that was correlation value between item score and construct score. The reflective indicator measurement indicated a change in an indicator in a construct if another indicator on the same construct changed (or was removed from the model). In Table 3, it could be seen that the indicator contained in the table was a valid indicator representing the construct that had a loading factor value > 0.5, while other indicators that did not exist in the table having a value of loading factor <0.5 had been removed from the model. Convergent validity was shown by the Average Variance Extraction (AVE) value. In Table 3, it was found that the AVE value for all variables was more than 0.5; it could be stated that all the variables and indicators of this study were valid. In the reliability test, the results of the research output showed that the value of composite reliability for all constructs was above 0.7, it indicated that all constructs in the estimated model met the criteria of discriminant validity so that it could be said to be reliable. This reliability test was reinforced by Cronbach's Alpha value indicating that all constructs were above 0.6. Thus all variables reinforced the latent variables or they were able to measure the latent variables.

#### 3.2.2. Result of Structural Model Evaluation

The evaluation of the structural model involved model capability testing and relationship between constructs [32]. The structural model in the PLS was evaluated using R^2^. The R^2^ value was used to determine the variation level of the independent variable changes toward the dependent variable. The R^2^ value can be seen in Table 4.

Based on the result in Table 4, it could be seen that the R-square value of the habit variable was 0.10. It could be interpreted that the habit variable was affected by the independent variables that were hedonic hunger and health interest was 10.2%, while the rest was affected by another variable. For habit variable, the R-square value was 0.009. It could be interpreted that sodium variable was affected by hedonic hunger variable, health interest and habit by 0.9%, while the rest was affected by another variable which did not exist in this research. Based on R-square value, the predictive relevance value, which could be used to know how the model was able to explain the information contained in the data, could also be calculated. The calculation of the predictive relevance could be done by using the following formula:

(1) Q2=1−1−R121−R22Q2=1−1−0.1021−0.009Q2=1−0.8980.991Q2=0.911=91.1%

The result of Q^2^ calculation indicated a value of 0.911. It meant that the model used in this study could explain the data information by 91.9%; the value indicated that the model was sufficient to describe the research problems.

#### 3.2.3. Hypothesis Testing

**Figure 1:**
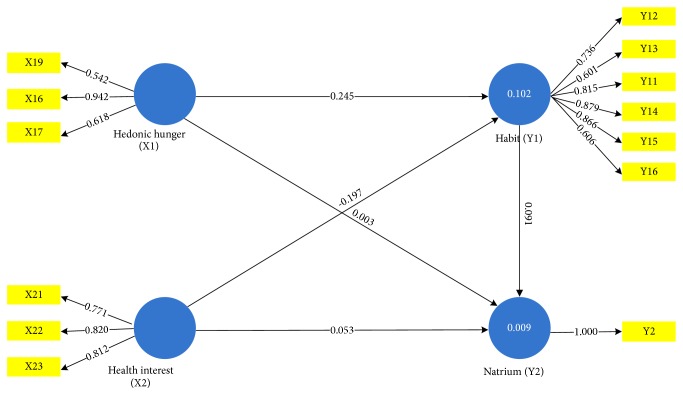
Research Path Diagram.

Hypothesis testing was done by using a resampling bootstrap method. Hypothesis testing was performed by looking at the calculated t value, if the calculated t value >1.98 at 5% significant level, then it could be concluded that the hypothesis was significant. The results of hypothesis testing can be seen in Table 5 and Figure 1

Table 5 gives the results of the calculated model coefficients; it can be seen that hedonic hunger positively and significantly affect habit with coefficient value of 0.245 and calculated* t* value of 2.052 which is greater than the calculated* t*. Health interest has a negative effect with the coefficient of -0.197, and it was significant with the* t* value of 2.236. Hedonic hunger had no significant effect on sodium with coefficient value of 0.003 and calculated* t *value of 0.024. Health interest had a positive effect but not significantly influencing sodium with coefficient value of 0.053 and calculated* t *value of 0.510. Even though showing a positive effect, habit has no significant effect on sodium with coefficient value 0.091 and calculated* t* value of 0.894. Since the habit did not have a significant effect on sodium, habit was not included in the mediation variables. The mathematical model derived from the path diagram was Y_1_= 0.245 X_1_ - 0.197 X_2_ and Y_2_ = 0.003 X_1_ + 0.053 X_2_ + 0.091X_3_.

## 4. Discussion

The result revealed that the average sodium consumption of 117 students of Universitas Brawijaya was 2.49 gram/day (Table 2). Sodium consumption at this level was above the level recommended by [31] which was less than 2 gram/day. The Ministry of Health also recommended consuming no more than 2.3 gram of sodium per day. High salt intake of the students was caused by their inability to control the source of their food.

The results of hypothesis testing showed that hedonic hunger and health interest had a significant effect on habit, but habit was inversely related to the interest of health. The respondents studied in this study were students and their dietary habit tended to choose fast food. This was consistent with a research conducted by [14] who explained that people still buy fast food even though their intentions are different. This behavior is difficult to change due to the long-standing habits. College students also had higher education, but it did not have a positive impact on health interest toward habit. [33] argued that education is an important socioeconomic factor in determining the risk of poor food intake, but another study conducted by [34] has shown that knowledge does not necessarily make a person behave in a healthy manner.

Hedonic hunger, health interest habit did not have a significant impact on sodium consumption. Respondents had a tendency to eat because of desire and pleasure, not because they wanted to meet the intake of nutrients in the body. This result supported the research conducted by [12] which showed that when hedonic hunger is paired with habit, it does not affect daily salt intake that gives an appetizing effect on food. Hedonistic behavior also caused the consumer to make too many mistakes when choosing food due to their inability to control the nutrients intake in food consumed [20].

Research also showed that consumers are not willing to compromise on the taste for the sake of health [22]. Consumers were still concerned with taste rather than health in choosing various foods such as the selection of corn chips [35] and functional foods [36]. It makes consumers ignore the nutritional content and nutrients intake in the body. [37] also described that health labels or logos such as “reducing salt” or “healthy choices” aim to facilitate healthy food choices for consumers, making it a warning to consumers who worry more about the taste of a product rather than health.

The habit of adding salt when cooking is a part of the culture of Indonesian society. This habit does not control sodium intake. People develop the habit of eating food because they repeatedly eat the same kind and amount of food in the same way [4, 38, 39]. [40] explained in his research that daily dietary consumption determines the healthy nature of a diet.

It should be noted that this study was limited on the 117 sample students within the University. Therefore, the results will only be applicable to the specific sample population, which is Universitas Brawijaya. Further research might be expanded with a larger sample size of University students.

## 5. Conclusion

Based on the research results, hedonic hunger and health interest have significant impact on habit but both have no significant influence on sodium intake. However, the effect of health interest was inversely related to health. Fast food eating habit and various choices of food with appetizing flavors could make consumers ignore the adverse effects of food on their health. The respondents had a tendency to eat based on desire and pleasure rather than fulfilling their required nutritional intake and therefore salt intake was not well controlled. Habits of eating salty food may result in ignorance on health and nutritional aspects.

## Figures and Tables

**Table 1:** Variable and Research Item.

Variable	Item	Notation
*Hedonic hunger* (X_1_)	I cannot resist eating delicious food when I see it	X_11_
	I worry if my mind concentrates on food	X_12_
	I feel so excited right before I taste my favorite food	X_13_
	I really want to eat something when I hear people talk about delicious food	X_14_
	It is hard for me to resist eating certain food even though I know that it is unhealthy	X_15_
	Sometimes when I do my daily activities, I want to eat something that crosses my mind all of sudden.	X_16_
	I get more satisfaction when I eat	X_17_
	Before I eat my favorite food, my mouth starts to be watery	X_18_

	When I eat a good food, I am very focused on the taste of the food	X_19_
	It is important to me that the food I eat must be delicious	X_110_
*Health interest* (X_2_)	I am very conscientious about the health aspects of the food I eat	X_21_
	I always adhere to health and dietary balance	X_22_
	It is important for me that my daily diet contains lots of vitamins and minerals	X_23_
	It is important for me that the food I eat contains lots of nutrients	X_24_
	It is important to me that the food I eat contains high protein	X_25_
Habit (Y_1_)	I love salty food	Y_11_
	I love salty snacks	Y_12_
	When I eat and the food not salty, I add more salt	Y_13_
	It is very difficult for me to avoid salty food	Y_14_
	Eating salty food is an act I do without thinking	Y_15_
	I eat salty food before I realize it	Y_16_
	I try consciously not to eat excessive salty food	Y_17_

**Table 2:** Description of Respondents' Characteristics.

Respondents Characteristics	Mean and Standard Deviation of Sodium Consumption (gram/day)
Gender	
Male	2.49 (1.36)
Female	2.46 (1.37)
Age	
16-20	2.76 (1.64)
21-25	2.37 (1.22)
Monthly Expenses of Participants	
< 1 million rupiah	2.52 (1.46)
1-5 million rupiah	2.40 (1.17)
Body Mass Index (BMI)	
Underweight (< 18.5 kg/m^2^)	2.24 (1.20)
Normal Weight (18.5-24.99 kg/m^2^)	2.55 (1.40)

**Table 3:** Average Variance Extracted (AVE), Composite Reliability, Cronbach's Alpha.

Construct	Item	Loading	Average Variance Extracted (AVE)	Composite Reliability	Cronbach's Alpha
*Hedonic hunger* (X_1_)	X_16_	0.94	0.52	0.76	0.61
	X_17_	0.62			
	X_19_	0.542			
*Health interest* (X_2_)	X_21_	0.77	0.64	0.84	0.72
	X_22_	0.82			
	X_23_	0.81			
Habit (Y_1_)	Y_11_	0.82	0.58	0.89	0.85
	Y_12_	0.74			
	Y_13_	0.60			
	Y_14_	0.88			
	Y_15_	0.87			
	Y_16_	0.61			
Sodium (Y_2_)	Y_2_	1.000	1.000	1.000	1.000

**Table 4:** R Squared Value.

Variable	*R Squared*
Habit (Y_1_)	0.102
Sodium (Y_2_)	0.009

**Table 5:** Hypothesis Testing Result.

Static Hypothesis	Path	Calculated t	t Table	Note
*Hedonic hunger* (X_1_)→*Habit* (Y_1_)	0.245	2.052	1.98	Significant
*Health interest* (X_2_)→*Habit* (Y_1_)	-0.197	2.236	1.98	Significant
*Hedonic hunger* (X_1_)→Sodium (Y_2_)	0.003	0.024	1.98	Not Significant
*Health interest* (X_2_)→Sodium (Y_2_)	0.053	0.510	1.98	Not Significant
*Habit* (X_3_)→Sodium (Y_2_)	0.091	0.894	1.98	Not Significant

## Data Availability

Herewith I declare that the quantitative data used to support the findings of this study are included within the article.
